# COVID-19 associated mucormycosis - a recent challenge

**DOI:** 10.4322/acr.2023.427

**Published:** 2023-04-13

**Authors:** Vitorino Modesto dos Santos, Lister Arruda Modesto dos Santos, Taciana Arruda Modesto Sugai

**Affiliations:** 1 Hospital das Forças Armadas (HFA), Department of Medicine, Brasília, DF, Brasil; 2 Universidade Católica de Brasília, Medical Course, Brasília, DF, Brasil; 3 Instituto de Assistência Médica ao Servidor Público Estadual (IAMSPE), Advanced General Surgery and Oncosurgery, São Paulo, SP, Brasil; 4 American Society of Neurophysiology, Brasília, DF, Brasil

Dear Editor,

Mucormycosis has been considered an uncommon, life-threatening infection by filamentous fungi living in the soil and vegetable or animal-decomposing matter, causing the infection through superficial lesions and spore inhalation or ingestion, mainly in people with diabetes, malignancy, hematological diseases, or immunosuppression condition.^1-12^

We read the report by Bhowmik et al.^2^ about gastric mucormycosis incidentally diagnosed in the autopsy of an Indian man who died of septicemia one week after a road traffic accident. Gastrointestinal (GI) mucormycosis is estimated to occur in up to 13% of the invasive infections, involving the stomach in 58% of the cases, or the small and large intestines. However, only one-fourth of these cases have been clinically diagnosed.^2^ The authors emphasized the incidental diagnosis of this life-threatening mycosis by endoscopy or autopsy, which has increasing in frequency in recent decades.^2^ The mortality rate of GI mucormycosis is up to 78% of cases, with perforated ulcers and peritonitis being the leading causes of death.^2^ This letter aims to present a review of this issue, including articles from 2020 to 2023.

Gurjar et al.^1^ reported a 22-year-old Indian woman who had acute pancreatitis two months after the normal delivery and evolved with thrombosis of the left cavernous venous sinus, diabetic ketoacidosis, and GI bleeding due to a large gastric perforation. Gastric samples revealed broad aseptate and foldable fungal hyphae and angioinvasion. She received intensive care support and underwent a laparotomy with subtotal gastrectomy, feeding jejunostomy, and ileostomy, in addition to amphotericin B and oral posaconazole. Notwithstanding, her clinical condition worsened, and she succumbed after six weeks. The authors emphasized the association of mucormycosis with diabetic ketoacidosis.^1^ Khsiba et al.^7^ reported fatal invasive gastric mucormycosis in a 61‐year‐old diabetic Tunisian woman and a previously healthy 59‐year‐old foreign man living in Tunisia. The woman had a one-week history of vomiting after 4 months of inhaled corticoids for cough, and the upper GI endoscopy with biopsy revealed invasive mucormycosis by *Rhisopus arrhizus*. The patient had to stop conventional amphotericin B due to a renal function impairment; she also underwent total gastrectomy 2 months after the diagnosis but died 10 days later.^7^ The male patient presented with gastric pain, vomiting, worsened general state, and fever; the upper GI endoscopy showed a bleeding gastric ulcer, and the biopsy study detected typical zygomycetes hyphae. He did not undergo specific treatment for mucormycosis, and the outcome included septic shock and hemophagocytic syndrome, dying after eight days of admission.^7^ The authors stressed the mortality being up to 85% without early diagnosis and therapy.^7^ Noor et al.^10^ reported a 21-year-old man presenting gastritis-like symptoms, who was recently treated for hemophagocytic lymphohistiocytosis, and a fungal sinusitis. The upper GI endoscopy revealed an extensive gastric involvement by mucormycosis, and the patient underwent antifungal drugs and resection of the necrotic gastric tissue. In this case, it was worthy of note the young age, the lack of risk factors such as diabetes or immunosuppression, and the development of hemophagocytic lymphohistiocytosis. The authors highlighted the role of early diagnosis and prompt treatment on outcome.^10^ Yuvaraj et al.^12^ described a 53-year-old man with diabetes mellitus and alcoholism who had acute abdominal pain and hematemesis. The imaging studies showed a gastric wall thickening and exophytic ulceration involving the greater curvature and the splenic hilum, and evidence of splenic infarction. Biopsy studies of the mass revealed broad aseptate branched fungal hyphae consistent with the diagnosis of gastric mucormycosis, and amphotericin-B was administered; however, the gastrectomy and splenectomy procedures were not performed because of the patient’s clinical condition, and he irreversibly evolved to death.^12^ The authors highlighted the high index of suspicion and physicians’ awareness needed for early diagnosis and treatment of mucormycosis, mainly in immunocompromised patients.^12^ Recent articles on COVID-19-associated mucormycosis (CAM) have been published.^3-6,8,9^ It is estimated a higher prevalence of CAM than the reported cases because of the lack of population-based research. Thus, CAM cases may be higher than the described ones.^4^ Patients with COVID-19 treated with intensive care support are prone to nosocomial and ventilator-associated infections.^4,13^

Chauhan et al.^3^ reported a 35-year-old man with fever, vomiting, delirium, dyspnea, and cough, in addition to melena. Pulmonary thromboembolism and bilateral ground glass opacities were revealed by chest imaging study. The upper GI endoscopy showed multiple gastric black ulcers. Histopathological examination of the gastric biopsy revealed a broad, classical aseptate, ribbon-like, and foldable pattern of the mucormycosis fungal hyphae. Under amphotericin B therapy, he presented mental confusion, paraparesis, and left upper limb weakness. Contrasted computed tomography showed ring-enhancing brain lesions that were considered as a manifestation of the yeast infection. Due to clinical suspicion of recent infection by SARS-CoV-2, the IgM test was positive. Treatment of this patient included posaconazole evolving to an asymptomatic clinical status and complete vanishing of the CNS lesions.^3^ Worthy of note is the disseminated mucormycosis in the absence of any apparent risk factor, except for COVID-19 infection that has been associated with prothrombotic conditions.^3^ Malakar et al.^8^ described an 82-year-old man with GI bleeding for five days who utilized prednisolone for 21 days to treat moderate COVID-19 infection 2 months ago. The upper GI endoscopy detected a gastric ulcer extending from the gastroesophageal junction, and biopsy studies showed fungal hyphae consistent with gastric mucormycosis. For 13 days, he used pantoprazole and amphotericin B, which was changed by posaconazole due to worsening renal function and continued for three months till the ulcer was healed.^8^ The authors emphasized a rare cause of GI bleeding in a patient who had COVID-19, and the extensive mucormycosis successfully treated by anti-fungal therapy without surgery.^8^ Monte et al.^9^ described an 86-year-old man presenting with acute diarrhea, cough, dyspnea, and fever five days before, and a throat swab confirmed COVID-19.^9^ With acute respiratory failure and hemodynamic instability, he had intensive care support and underwent a schedule of ceftriaxone, azithromycin, oseltamivir, and hydrocortisone. Five days later, he had melena, and the upper GI endoscopy showed two giant gastric ulcers in greater and lesser curvatures; a histopathological study confirmed mucormycosis, but the patient died one week following admission and before the established diagnosis. The authors cited preemptive therapy in a patient with features suggesting mucormycosis.^9^ Dam et al.^4^ reviewed the pathogenesis, causal agents, determinants, distributions, manifestations, virulence factors, case reports, diagnosis, and treatment related to the increasing trend of mucormycosis in several countries and the COVID-19 co-infections. The study comprised 388 cases and showed 46.7% of global mortality; 56.8% of the cases were associated with uncontrolled diabetes and 10.2% with trauma, the majority of cases and the higher mortality rate were observed in North India (82.7%).^4^ GI infection was uncommon and with nonspecific symptoms, affecting the large intestine (∼43.2%), stomach (∼33%) often in adults, and small intestine (∼28.4%), mainly in children. The mortality was 96% in disseminated, 85% in GI, and 76% in pulmonary infections.^4^ The authors stressed the risk of improper use of antifungal drugs for the mucormycosis surge in the present pandemic, which might contribute to antifungal resistance over time.^4^ Didehdar et al.^5^ reviewed 87 reports of GI mucormycosis from 2015 to November 2021, including 70 adults and ten neonatal patients, the majority of cases (57.5%) from Asia. The mortality rate of neonatal patients was 70%, the other cases had a mortality rate of 44%; corticosteroid use and diabetes were the main risk factors, 11% were immunocompetent, and four of the adults presented with specific positivity tests of COVID-19 infection.^5^ Abdominal pain, fever, and perforation were common; 40% of the neonatal cases presented vomiting. Surgery plus antimycotic agent (61%), antimycotic therapy alone (28%), and surgery alone (11%) were the therapeutic approaches. All neonatal patients underwent a surgical procedure; posaconazole (30%) and isavuconazole (11%) effectively treated these patients.^5^ The authors commented on low immunity during the COVID-19 pandemic favoring the high prevalence of mucormycosis in India and the difficulties of early diagnosis and treatment.^5^ Divakar^6^ reviewed data about mucormycosis in general and the cases associated with the two waves of COVID-19 infections in India.^6^ The estimated mortality rate of mucormycosis (50%) may be over 90% if untreated; *Rhizopus arrhizus* was the most common agent of human mucormycosis described in Indian patients, followed by *Apophysomyces variabilis*, *R*. *microsporus*, and *R*. *homothallicus*.^6^ Mucormycosis among COVID-19 patients, including the mortality rate, are increasing in India and some of the neighboring countries such as Bangladesh, Nepal, and Pakistan.^6^ Because of species-specific differences, there are therapeutic challenges in India, with only three potentially efficient drugs (amphotericin B, posaconazole, and isavuconazole). The authors cited accurate species identification as crucial for the correct diagnosis of mucormycosis, and for developing species-specific antifungal drugs.^6^

In conclusion, although considered a rare entity, GI mucormycosis must be a plausible hypothesis if an atypical gastric ulcer is detected in people with COVID-19.
